# Behavioural approaches and conservation messages with New Zealand’s threatened kiwi

**DOI:** 10.1016/j.gecco.2021.e01694

**Published:** 2021-08

**Authors:** Patrick J. Walsh

**Affiliations:** a Manaaki Whenua-Landcare Research, 231 Morrin Road, Auckland 1072, New Zealand; b US Environmental Protection Agency, 1200 Pennsylvania Ave, NW, Washington, DC 20460, USA

**Keywords:** Randomised control trial, Nudge, Behavioural intervention, Conservation, Threatened species

## Abstract

New Zealand’s iconic bird species, the kiwi, is facing significant threats from dogs. Dogs walked off leash or left outside frequently kill fragile kiwi, posing a major social issue. Local governments have spread awareness through billboards, pamphlets, and other media, but there are no empirical analyses of message effectiveness or targeting. There is a dearth of research on behavioural interventions in conservation, where pro-environmental behaviour is typically costly to individuals. This study uses a randomised control trial (RCT) to test the effectiveness of four different messages on a local policy for dog registration, using thousands of households. The RCT aims to increase dog registration fees, which, although compulsory, have low compliance in some areas. Results suggest considerable heterogeneity in response across messages, with only messages focussed on kiwi conservation and dog attacks having an impact on registration, and only in some groups. A social norm /”nudge”-based message had no effect. Results should help design future messaging programs, as well as raise funds for conservation through additional registration fees. This study is one of the first to use an evidence-based approach for conservation messages in a critical area and provides several implications for future policy and engagement campaigns.

## 1. Introduction

Kiwi are facing significant threats from dogs and other mammals in Northland, New Zealand, with an average life expectancy of almost half that of other regions. Kiwi lack a breastbone and have unique, strong smells, so that dogs seek them out and can easily kill them, even with minor bites. Research from the Department of Conservation (DOC) finds that dogs are the largest threat to adult kiwi.^1^ In Northland it is common to walk dogs off leash and some owners allow their dogs to wander at night.^2^ Uncontrolled dogs are also a threat to other forms of wildlife, including marine animals and other birds and native species (Banatoski et al., 2017). A common response to this persistent problem is to spread awareness through billboards, pamphlets, and other media. However, there is not much evidence-based research on message effectiveness in conservation, and there are reports of declining responses to messages featuring the kiwi.^3^

After years of struggling with kiwi population numbers, there is broad interest in using new tools to assist conservations efforts, such as nudges and other behavioural insights (Bennett et al., 2017).^4^ These non-regulatory tools have been used to obtain better outcomes in several areas, including energy use (Allcott, 2011; Jachimowicz et al., 2018), water use (Bernedo et al., 2014), and land management (Byerly et al., 2018). However, in many previous studies, individuals are “nudged” into behavior that both benefits them personally and is better for the environment. For example, promoting more efficient energy use saves the consumer money and is better for the environment. There are few studies that evaluate conservation behaviour, where pro-environmental behavior can place significant costs on the individual (both in terms of money and/or time) (Kidd et al., 2019).

This paper describes an experiment that evaluates conservation-related behavioural interventions with dog owners. Although there are many responsible dog owners in Northland, it is necessary for some to change their behavior and habits in order to help protect local threatened wildlife (James, 2001; Dale et al., 2013). Because the factors that will help protect Kiwi - keeping dogs inside at night and leashed on walks, and increased kiwi aversion training - directly result from owner behaviour, there is an opportunity to test the efficacy of nudges and behavioural insights in influencing owner behaviour.

There is also a lack of evidence about the impact of conservation messages on different groups of people, in order to get the most effective message to the most receptive group (Kidd et al., 2019). Northland is a diverse and larger part of New Zealand, with notable differences between urban, rural, and agricultural areas.^5^ Different populations and areas may respond in dissimilar ways to conservation messaging. To obtain the most impact from conservation messages, potential heterogeneous responses must be investigated.

**Fig. 1.**
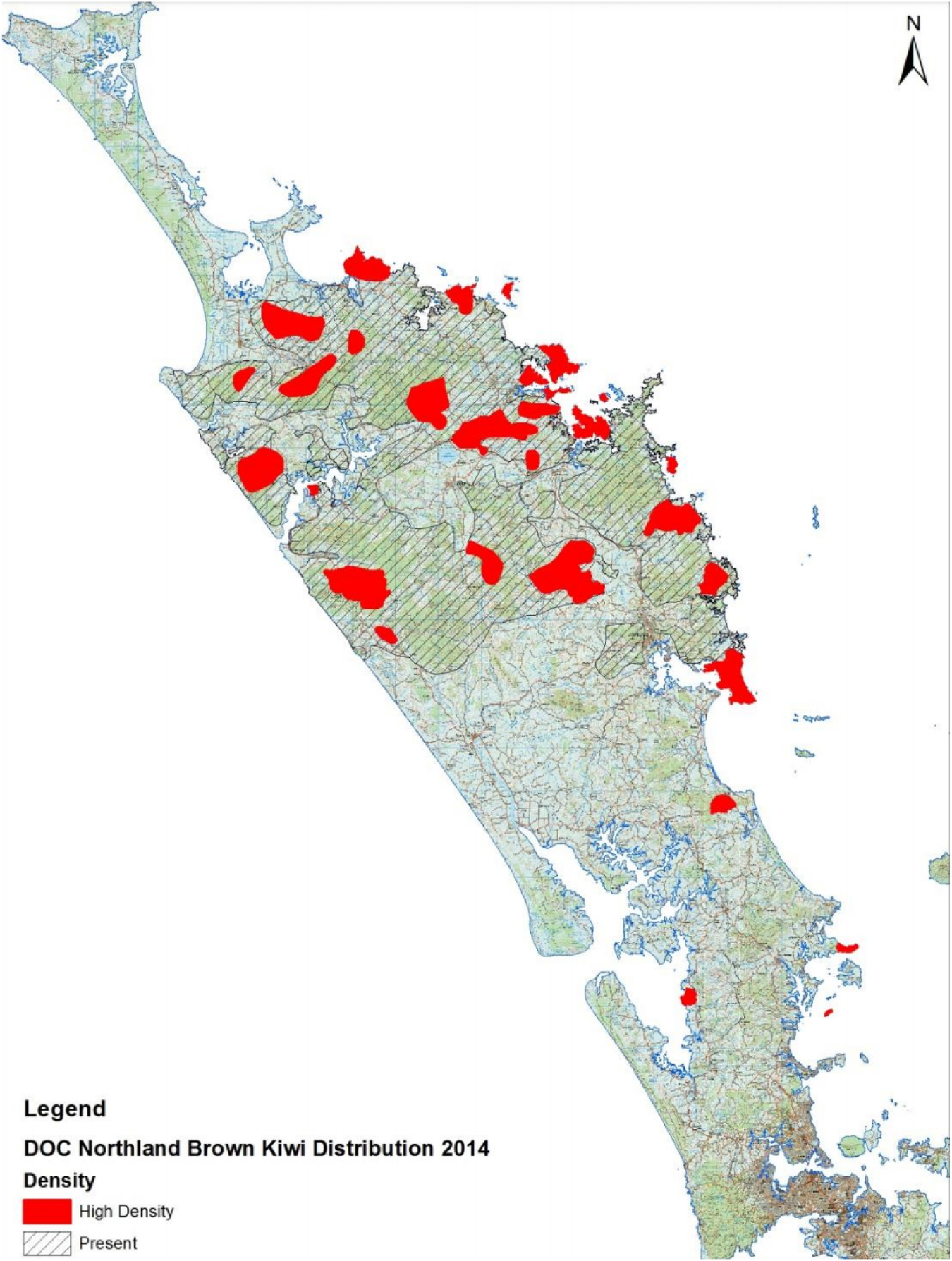
Northland Kiwi habitat.

A randomised control trial (RCT) was instituted in partnership the Far North District Council, (FNDC) a local government with a large kiwi population in its area (Fig. 1). Residents are required to register all dogs annually, with registration fees helping to fund animal control. However, compliance rates are quite low in some areas, so the council was interested in exploring behavioural approaches for increasing registration rates. With more fees paid, animal control activities could be better resourced. In an annual mail reminder, the RCT deployed four conservation-related messages aimed at improving registration rates. These messages were based on past literature (St John et al., 2010; Allcott, 2011; Allpress, 2019; Kusmanoff et al., 2020) and local priorities. They focussed on kiwi protection, dog attack prevention, a behavioural nudge, and dog loyalty.

The RCT allowed an empirical test of conservation-related messages in a domain with real consequences using over six thousand households. The households in the sample can have a real impact on wildlife conservation through their actions since their registration fees fund animal control, so it is important to identify which messages motivate them. Although the RCT was conducted in northern New Zealand, the nature of the problem mirrors many similar conservation issues worldwide. For instance, there is an established link between local human behavior and wildlife that is regularly highlighted in local media, and yet the behavior persists.^6^ There is also significant interest in protecting the species of concern—the kiwi is a national icon.

## 2. Background and literature review

There is significant interest in dog control in Northland because of the prevalence of kiwi kills and dog attacks on people. The local and national news regularly report on kiwi killed by dogs and the problem of uncontrolled dogs has become a polarising local issue.^7^ Data from the New Zealand Government shows an average of 376 dog attacks from 2015 to 2019 (to people) per year in the Far North District (costing an average of $90,000 - $120,000 in health costs per year), yielding the third highest per capita rate in New Zealand districts.^8^ A recent report (Kannemeyer et al., 2019) explored Northland-based viewpoints on dog control and kiwi conservation using surveys and Q-methodology. Kannemeyer et al. noted several barriers to local behavioural change, such as favoring pets over wildlife. Although recognizing the importance of kiwi, some people were unwilling to accept limits on dog ownership (e.g. no more than two dogs) in developments near kiwi habitats.

**Fig. 2.**
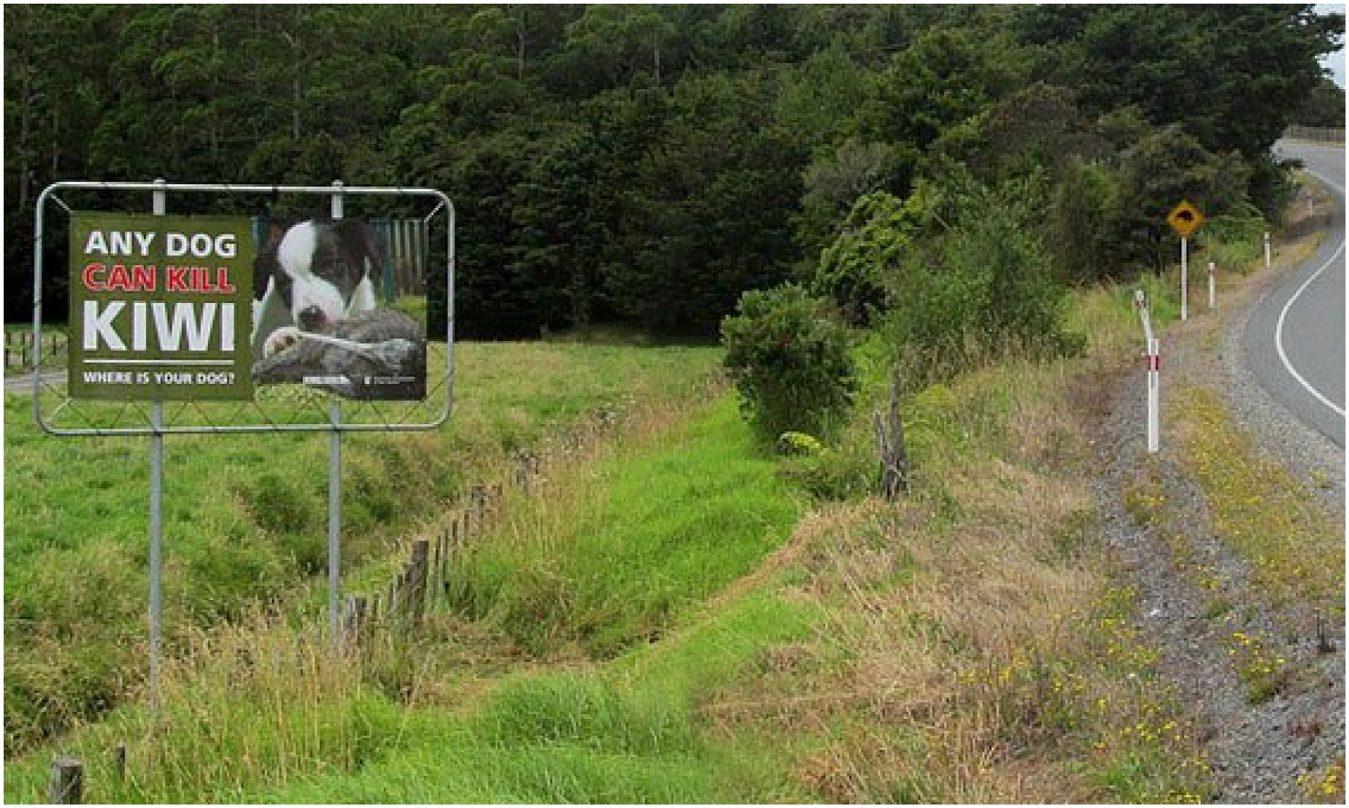
Example kiwi message signs. https://www.doc.govt.nz/news/media-releases/2013/new-kiwi-road-signs-in-the-whangarei-area/. Source: Kiwi coast, Department of conservation.

**Fig. 3.**
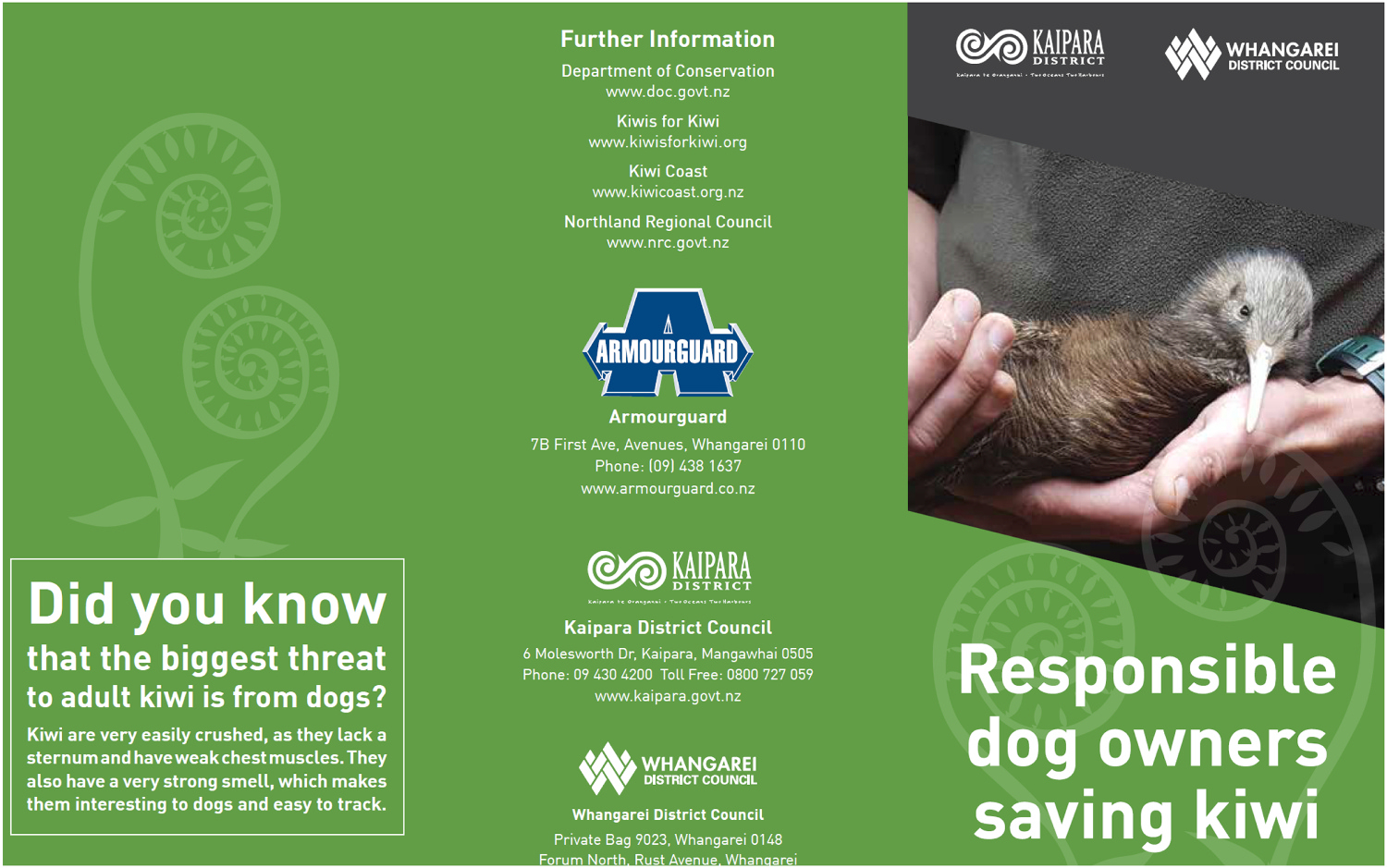
Kiwi pamphlets. Source: Kiwicoast

Several previous efforts have tried to spread awareness about kiwi-dog interactions (Fig. 2 and Fig. 3), but it is not clear if they are having any impact.^9^ There is therefore a desire to evaluate the effectiveness of messaging campaigns in order to help local government activities and increase revenue for dog control activities. Since their actions can have significant impacts on kiwi, dog owners are the main targets of this experiment.

Human behavior plays an essential role in many environmental problems, and there is a rapidly expanding literature that explores inexpensive strategies to shift behavior (Allcott and Rogers, 2014; Byerly et al., 2018). These types of tools are important in conservation programs with local governments, where funding is limited, and enforcement can be difficult. The behavioural approach (Thaler and Sunstein, 2008) aims to take advantage of insights from psychology and related fields that emphasize the role of biases and cognitive constraints in peoples’ choices (Byerly et al., 2018). For instance, changing the default setting, or characteristics of a person delivering a message, can have a significant effect on outcomes (Kondylis et al., 2016). These unconscious influences can be used to “nudge” people into more socially optimal behavior (Fanghella et al., 2019). If these types of approaches can induce behavioural change in dog owners, there could be benefits for the council and for kiwi conservation.

In an early paper with a sample of over 600,000, Alcott (2011) found that amending utility bills to include messages about the electricity usage of neighbours could significantly lower energy use, inducing cost savings and climate benefits. Later follow-up research (Allcott and Rogers, 2014) confirmed initial results and indicated a lasting effect of the social norm-based messages. Other papers have shown the potential of behavioural interventions to improve environmental practices or attitudes, including applications in water use (Bernedo et al., 2014), environmental donations (Kubo et al., 2018; Fanghella et al., 2019), and energy conservation (Jachimowicz et al., 2018).

In the present application, new tools are needed to improve compliance in dog registration and to understand dog owner responses to messages. There are several options for behavior change, including approaches from the social marketing literature, such as commitment devices (Barr et al., 2011) and environmental prompts (Moussaoui et al., 2020). Previous behavioural studies have demonstrated the value of non-regulatory approaches to induce behavior change, with the potential of achieving better environmental outcomes at a low cost (Kormos et al., 2015), which are attractive features in the present setting. In some contexts, this is also preferable to a legislative approach, which can take significant time to accomplish.^10^

However, while there are several behavioural-focussed studies on environmental issues like sustainability, there are very few applications with a conservation focus. For kiwi conservation and dog registration-related behaviours, there are questions about the applicability of previous results. Kidd et al. (2019) note (p. 93) that “little is known about the influence of message design on biodiversity conservation behaviours.” Conservation-based behaviour change differs from other applications like sustainability in several notable ways, such as the cost imposed on the subject. For example, promoting reduced energy consumption (as in Alcott (2011)) can both save money on utility bills and improve the environment. In many conservation applications, the desired behavior can come at a significant monetary (or psychological) cost with little to no direct benefit to the individual. With kiwi-dog interactions, there is a prevalent culture of off-leash dogs, with complaints that leashing imposes a significant burden (Banatoski et al., 2017).

On the other hand, some people are willing to pay more for green energy, and changing the defaults on energy contracts has induced significant uptake in more expensive green energy (Momsen and Stoerk, 2014; Ebeling and Lotz, 2015). These papers point to potential heterogeneity in response across subjects, topics, and approach. Momsen and Stoerk (2014) find that the type of nudge was critical in renewable energy applications, with several proving ineffective.

There is also research suggesting that some groups that are less responsive to nudges. Goldstein el al. (2008) find that social norm nudges were more effective when they closely matched the behavior of focus. Costa and Kahn (2013) find that liberal households are much more responsive to social comparison nudges in energy saving than conservatives. Social norm messages about vehicle use was found to be much more effective for commuters than others (Kormos et al., 2015). It is therefore important to analyze differential responses to conservation messages, so that messages can be better targeted to local populations. Northland has wide variation in socio-demographic characteristics between urban and rural areas, as well as between the east and west (where much more tourism occurs). Differences in socio-demographics are associated with environmental preferences and beliefs more generally (Eppink et al., 2021).

There is one previous New Zealand-based paper on conservation behavior and cat ownership that yields potential local insights about message types. Outdoor cats can have significant impacts on local biodiversity (particularly New Zealand’s native birds), so MacDonald et al. (2015) surveyed people about keeping cats indoors. Each respondent was given one of several persuasive messages about indoor cats. Results suggested that messages focused on veterinarian recommendations and peer behavior were better received than messages about cat impacts on biodiversity. Although the paper was based on survey intercepts at a zoo instead of observing actual behaviour, it suggests there may be value in using complementary messages to indirectly motivate conservation behaviour.

In many cases, behaviour change is needed in areas where regulation already exists as a motivation. Off-leash dogs are already prohibited by councils in most areas of Northland. There are a variety of reasons that people do not comply with rules or best practices, with monetary, time, or psychological burden being only partial explanations. People may also be motived by anticipatory beliefs about health (Janz and Becker, 1984), or protection and stress (Rogers, 1985). A common response to compliance problems is to provide additional information about the problem, or consequences (Fig. 2 has a current Northland example). This relies on the theoretical concept of a “knowledge deficit” (Kidd et al., 2019), assuming that informing people will change their behavior, but many applications in conservation have fallen short (McLeod et al., 2015). Some existing efforts in Northland use this approach (Fig. 3).

## 3. Experiment and data

### 3.1. Experimental design

Every year Northland dog owners are required to register their dogs with their district council and pay a fee. Registration provides important data to the council, which helps plan animal control activities and supports animal health, sterilization, and other programmes. The registration database is also used to support the enforcement of legislation and reunite lost animals with owners.

Although registration is mandatory, typically only 60–70% of those who are mailed registration reminders in the Far North District actually register, so the council is interested in both promoting conservation and improving registration rates. Registration reminders are sent annually to all households that have previously registered, as well as other dog owners that the council is aware of (for instance, previously unregistered dog owners that are given dog-related citations). These annual registration reminders were identified as a vehicle to implement an RCT, which will allow a test of different messages on the registration rates of dog owners. This will give insight into behavioural responses from dog owners.

This RCT should provide key evidence to advance kiwi conservation. First, it is important to determine the effectiveness of different message types on motivating dog owners. These different messages may be receptive to different types of owners. Results can help design future awareness campaigns and better target campaigns to the most receptive areas and populations. Second, conversations with FNDC stressed a limited budget for animal control activities, such as capturing wandering dogs—which are a large threat to kiwi. Improving registration rates through messaging will provide additional funding for animal control.

Four separate messages were designed in partnership with the Council, local kiwi conservation advocates, and the Department of Conservation, to be randomly inserted in registration reminders that are mailed out to addresses in the Council’s dog database. Following the literature review, several concepts were emphasized in message development. There was a desire to use several different messages to account for potential heterogeneity in message effect (Goldstein et al., 2008). It is also important to have each message be straightforward and widely applicable (Thaler and Sunstein, 2008). For presentation, the messages were deployed alongside a bespoke graphic to appear more attractive.^11^ These four alternatives were focussed on the following themes:

1. Kiwi conservation
2. Dog attacks
3. Social comparison nudge
4. Dog loyalty

The kiwi message informed the recipient that dog registration can help plan kiwi protection and conservation activities, and that they are likely near kiwi habitat. This message should appeal to conservation-minded dog owners and should increase the salience of threats posed by dogs to kiwi. As emphasized by BIT (2015), this should bring more focus onto the immediate costs and benefits of their actions, especially with respect to kiwi. The other three messages did not directly mention conservation, but instead appealed to other motivations to promote the same action. Given the preference for non-biodiversity focussed messages in MacDonald et al. (2015)’s cat messaging study, it was deemed important to assess dog owner’s responses to complementary issues.

The second message focused on dog attacks. If people are not motived by kiwi attacks, they might be concerned with the more salient issue of dog attacks on people. This is intended to appeal to all dog-owners and is framed to emphasise the role registration plays in community wellbeing. This makes the message more social (BIT, 2015), and emphasizes community wellbeing and a commitment to others. In a messaging study in Chile, Villatoro et al. (2019) was also concerned with wandering dogs and found greater support for dog owner fines in areas with higher people and livestock-related dog attacks.

The third message was inspired by other behavioural-economic nudges (Bernedo et al., 2014; Allpress, 2019), and used a social-comparison nudge, informing recipients about the district-wide proportion of dogs registered. This message seeks to evoke a social norm of registration by emphasising the prevalence of owner-registration behaviour. Following similar literature in sustainability, a descriptive norm was chosen over an injunctive norm (the registration is already technically required by law). The figure cited in this message was based on the dog registration database for the previous year. The message was “nearly 8 out of 10 dogs in the Far North District whose owners receive this form are registered before penalties are applied.”

**Fig. 4.**
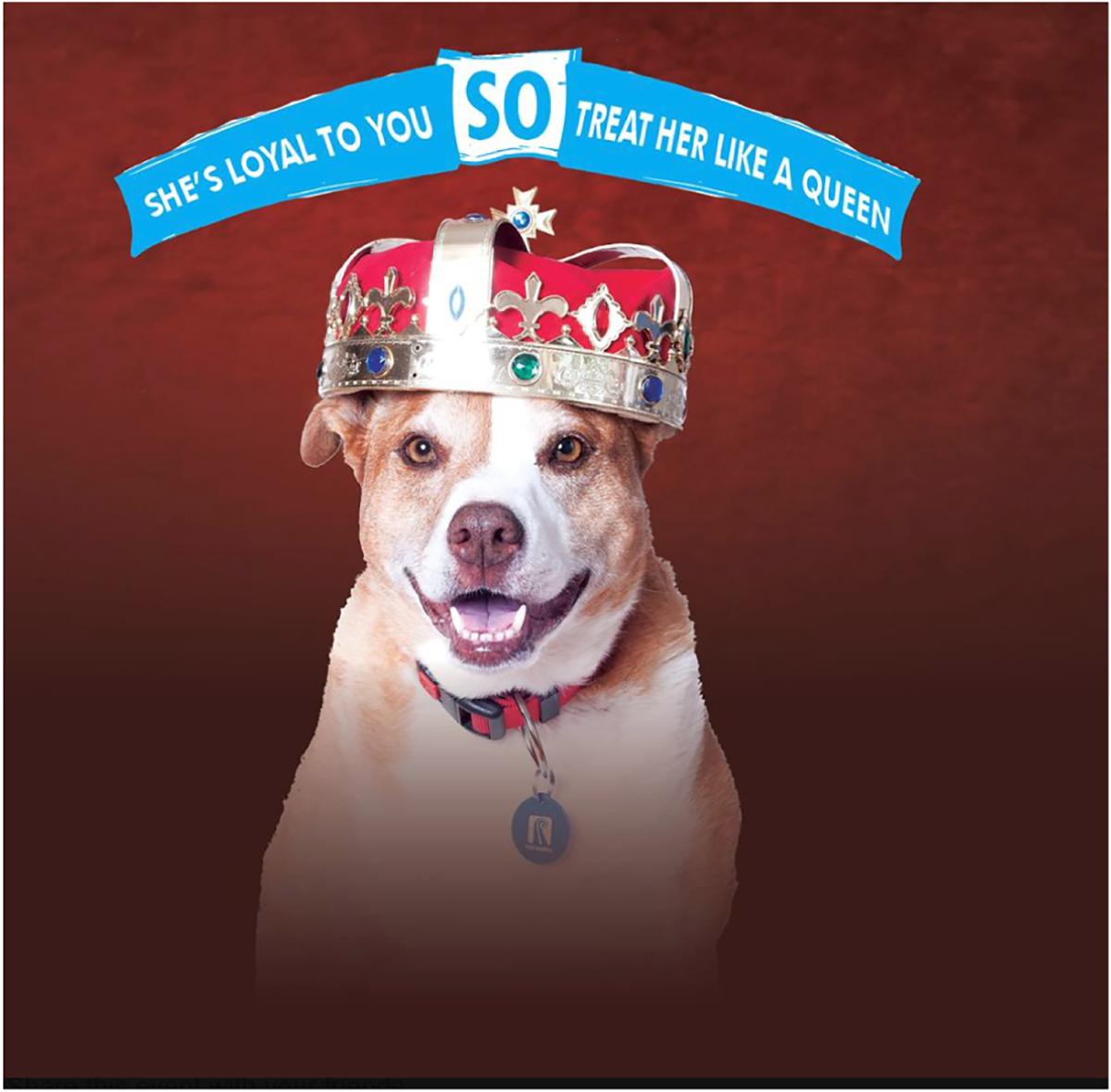
FNDC “loyalty” campaign flyer.

The final message was dog-focused, reminding people of their dog’s loyalty to them, and that a registered dog is more likely to be returned to them if it gets lost. This aims to change behavior by through people’s rational motivations. People may not like paying fines for registration but reminding them of the benefits and appealing to their emotional connection with their dog may be influential. Similar examples include pictures of lung damage on cigarettes (Christakis and Fowler, 2008; Haynes et al., 2012; Hallsworth et al., 2016; Congiu and Moscati, 2020). It also evokes reciprocity, and thereby creating in the owner a sense of acting in kind.^12^ Similar language was used by the council in a recent (highly successful) local animal control campaign. That campaign was designed with local residents using contemporary themes (for example see Fig. 4) and resulted in hundreds of new animals getting spayed/neutered.

**Fig. 5.**
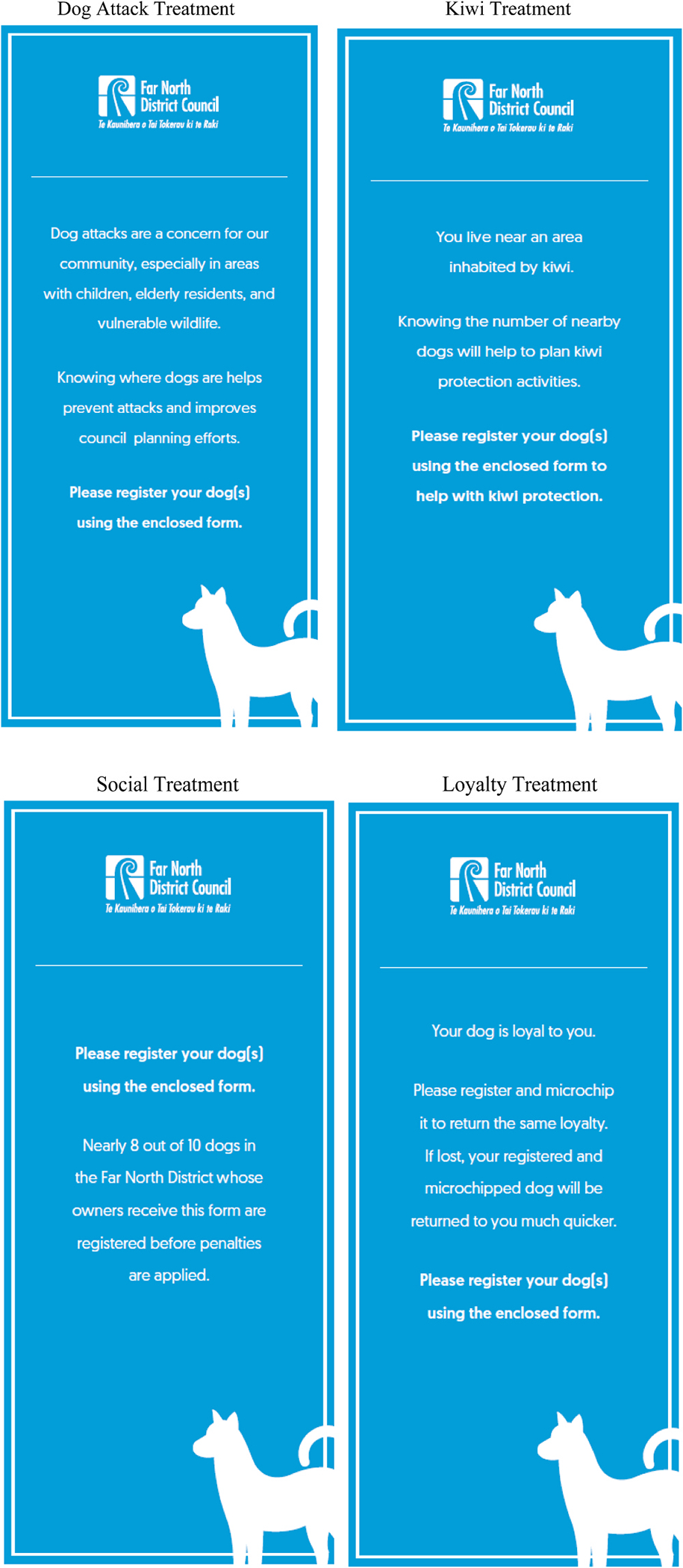
Dog registration messages.

The final set of formatted messages, designed with input from the council and an in-house graphic designer, appear in Fig. 5. These were printed and inserted next to the dog registration reminder form in the annual mailout.

Annual reminders were sent out to almost 7000 households, which were split between the four messages and the control group (no message). The council uses a local print shop to print, envelope, and distribute their annual reminders. They were provided with a spreadsheet for each zip code that randomly sorted addresses into one of the five groups (control + four treatments). The final design had 67% (2/3) of total households within a post code zone receiving a message, with 33% (1/3) of targeted households in the control group (no message).

#### Data.

**Fig. 6.**
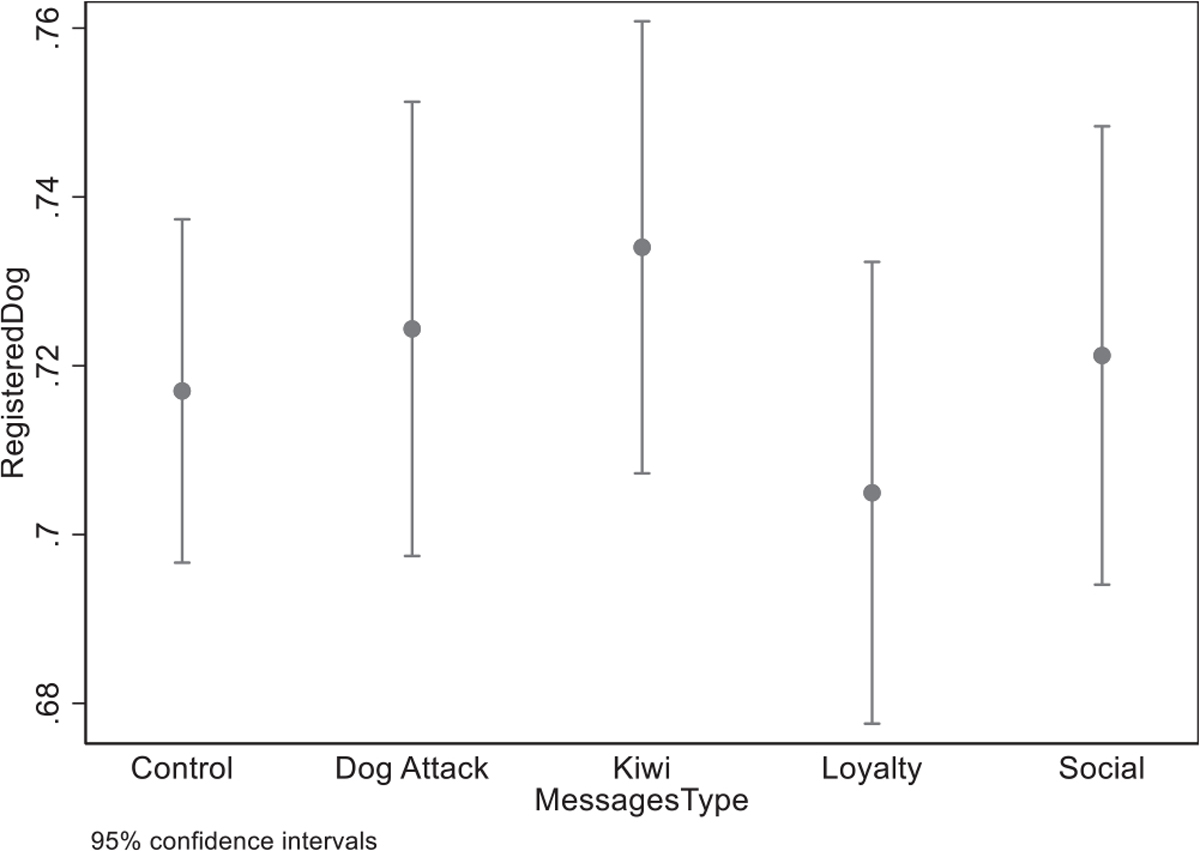
Mean and 95% confidence intervals of registration, by message.

The messages were sent out in July/August of 2018. Over the next year, registrations were recorded and matched with a database of owner IDs, yielding 6271 total responses.^13^ In the final sample, 4384 households received a message and 1887 did not. Table 1 contains a summary of the final message and control counts after data cleaning and merging. That table also shows the raw results of the registration by message. In the control group, 71.7% of people registered, while 72.1% of people who received a message registered. Looking at differences in registration across messages, the kiwi message sample had the highest registration rate, at 73.4%, while the loyalty group had the lowest registration rate at 70.5%. Fig. 6 plots the means and confidence intervals for the results by message.

Due to privacy concerns, there were unfortunately few characteristics in the data regarding owners. Although we do not know what exact postal codes households are in, we have a postal code ID number that identifies which households are in the same postal code. The council also provided data on registration from the previous year. However, attributes from the previous year were much more difficult to merge to current data.^14^ Table 2 summarises the data we have about dogs and owners. Approximately 20% of households have working dogs and 80% have pet dogs. The most common dog name is Max, with the most common breeds being Labradors and Labrador mixes (at 11.4% of the sample). The average household in the sample has 1.5 dogs, with some having as many as nine dogs. Using the data on dogs, it is possible to estimate the total cost of registration for each household (since prices are a function of these dog characteristics). The table also shows the postal code-level registration for 2018. Across all groups, registration was down in 2019 compared to 2018.^15^

To explore the success of the randomization, these summary statistics can be computed for each treatment (Table 3). The means and standard deviations of each variable are close across the different treatments. There are only minor differences in the summary statistics across the different treatments.

**Fig. 7.**
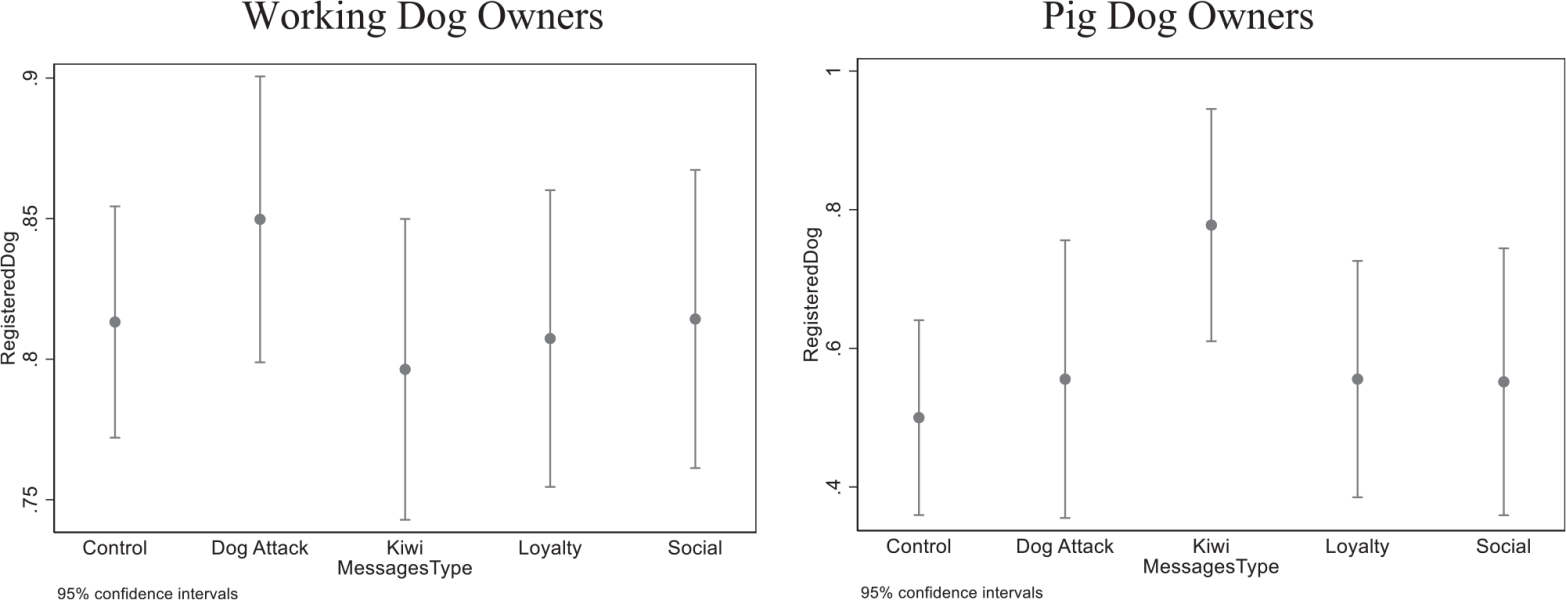
Mean and 95% Confidence Intervals of Dog Registration for Working Dog and Pig Dog Owners, by Message.

Previous studies suggest a heterogeneous response to some messages across different groups of people, so Table 4 show the registration results for working dog owners and pig dog owners. Working dogs are defined as working for the government, police, as a disability assist dog, or as part of a commercial activity (such as sheep dogs).^16^ Conversely, pig dogs are used for recreational pig hunting, which is a popular activity in many rural communities in Northland.^17^ Although the samples here are smaller, based on the raw data the Dog Attack message looks more successful with working dog owners, while the kiwi message appears the be very successful with pig dog owners (dogs that help with pig hunting). The control group of pig dog owners registered at a rate 48.1%, while households with a kiwi message registered at a rate of 77.8%, yielding a 60% increase in registration rates. Fig. 7 plots the mean and 95% confidence intervals of registration, by message types, for working dog and pig dog owners.

## 4. Methodology

To start analysing the experimental data, we are first interested in whether the receipt of the message had a statistically significant impact on the probability of registering. A basic econometric model of this relationship appears in Eq. (1), where Message_i_ indicates whether household *i* received a message, ***x*** is vector of other characteristics that affect registration, and β and γ are parameters to be estimated. To econometrically analyse this question, we use a probit model. Since we are also interested in the impact of the different messages, Message_i_ is a vector in the following equation.

(1) $$\mathrm{Pr}(\text{Register})=\Phi \left(\gamma Message_{i}+\text{xβ}\right)+\varepsilon$$

It is also important to control for other factors in the analysis to better identify variation in the probability of registering. For example, the total registration cost, age of the primary dog (first registered), and whether the household has work, pet, or pig dogs. Eq. (2) shows this relationship, where **Dog** is a matrix of dog-related characteristics specific to each household and **HH** is a vector of household characteristics.

(2) $$Pr(\text{Register})=\Phi \left(\gamma Message_{i}+\beta_{dog}Dog_{i}+\beta_{HH}HH_{i}\right)+\varepsilon$$

Although we don’t have households’ actual post codes, we have anonymized post code identifiers that allow identification of which households are in the same post codes. It is therefore possible to cluster standard errors at the post code level and use post code fixed effects.

There are also several reasons a household might fail to register, but not be violating the law, essentially showing up as a “false negative.” For example, dogs die or get lost and households move outside of the area. Ideally, we would remove these false negatives from the analysis, but they cannot be identified in the data. The incidence of this likely varies across two identifiable factors, which might also be useful for further targeting of messages.

I take two additional steps to test for additional variation in the effectiveness of messages. First, I use several interactions with the previous year’s post-code level registration rate. This can help identify areas that have the most potential “slack” in their registration rates, as well as control for places that might be at the maximum potential registration rates, given turnover/exit. Under the hypothesis that the previous year’s (post code-level) registration rate affects the impact of the different messages, I estimate the following specification:

(3) $$Pr(\text{Register})=\Phi \left(\gamma Msg_{i}+\beta_{PCode}PC_{i}+\beta_{PCMsg}PC*Msg_{i}+\beta_{\mathrm{dog}}Dog_{i}+\beta_{HH}HH_{i}\right)+\varepsilon$$

In Eq. (3), both continuous and non-continuous interactions are used for **PC**. Linear and quadratic versions of the 2018 post-code registration (PC) are interacted with the messages.^18^ I also split the registration rates into quartiles and interact quartile dummies with the different messages.

Second, I also explore additional specifications to test the hypothesis that dog age affects the impact of messages. If older dogs are more likely to die and hence result in false positives, owners of older dogs may be less likely to register. Similar to (3), specification (4) includes interactions with dog age-related variables.

(4) $$Pr(\text{Register})=\Phi \left(\gamma Msg_{i}+\beta_{PCode}PC_{i}+\beta_{AgeMsg}Age*Msg_{i}+\beta_{Age}Age_{i}+\beta_{HH}HH_{i}\right)+\varepsilon$$

Finally, several econometric models are used to test hypotheses related to message targeting. There are dog classifications (working dogs, pig dogs, and pet dogs), which could be relevant for message effectiveness and more general conservation activities. Pig dogs, for instance are let loose on pig hunts, and have the potential to come across kiwi burrows. It is therefore important to test whether they are responsive to conservation messaging to both get more pig dogs registered and to get them enrolled in other programs like kiwi aversion training.

## 5. Results

When econometric models are run on the overall sample, there is a positive but insignificant effect of receiving any message (Appendix Table 6). This suggests that simply receiving a message, no matter the content, does not have a significant impact. It is next important to test the impact of the different messages and different respondent characteristics.

To start exploring heterogeneous effects, the data are split into quartiles by the previous year (2018) post code-level registration rates, and dummies for those quartiles are interacted with the message indicators. This should help test whether the messages had different impact on different areas. To test the robustness of results several different specifications were estimated (Table 5) using alternate combinations of variables and fixed effects. Moving from left to right in the Table, additional control variables are added, with the final column including post code fixed effects. The results were fairly consistent across these specifications. Starting with the variables unrelated to messages, working dog and pig owners were less likely to register, all else constant. Total cost was negatively related to registration (as expected), and owners with more dogs were more likely to register.

For the impacts of messages, the fourth quartile with the highest previous registration rates was the omitted category, so the uninteracted message coefficients represent the message impacts in those areas. In the specifications with the full set of controls, (3 and 4), only the kiwi and dog attack-related variables were significant. Results indicate that those messages had a positive and significant impact on dog registration in most areas except for the fourth quartile, which saw a negative and significant impact.

**Fig. 8.**
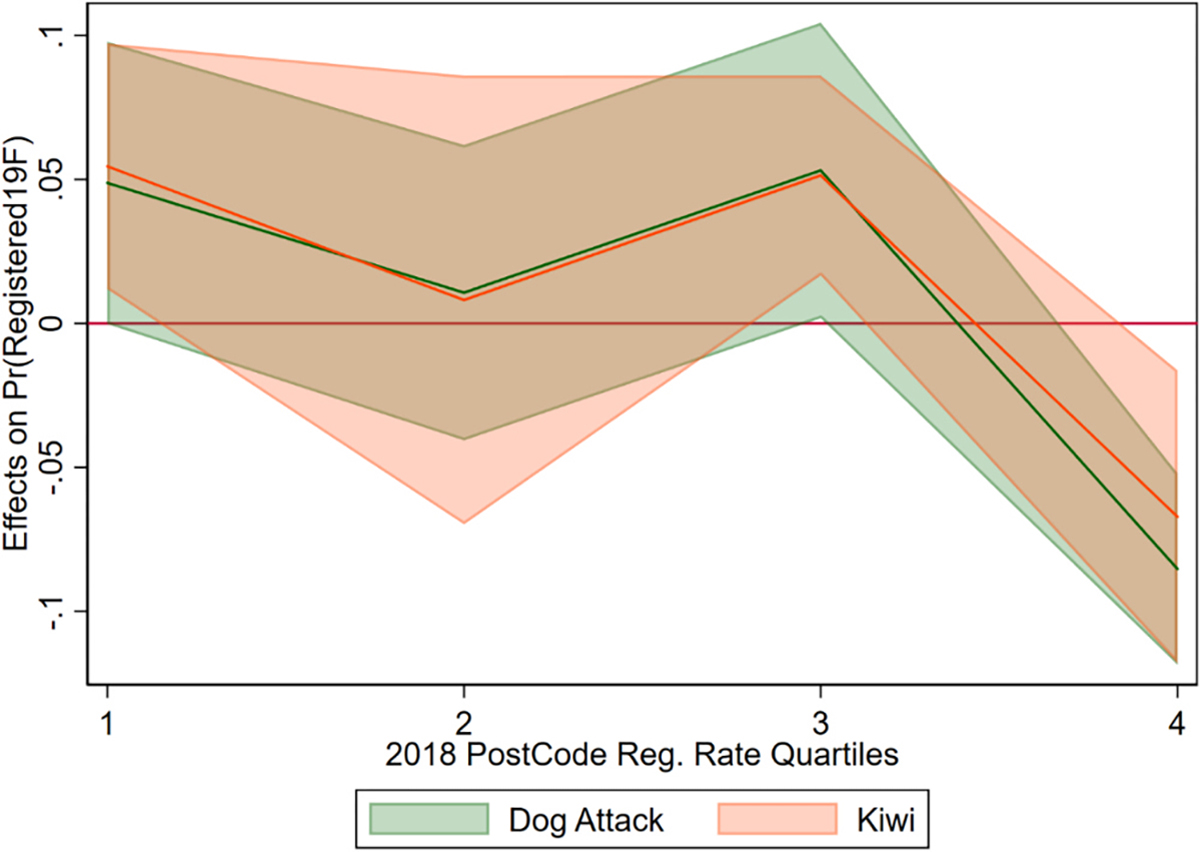
Marginal effect of kiwi message and dog attack message across registration quartiles.

To better depict the message effects, the marginal impact of each message is plotted for each quartile (Fig. 8). The messages had a positive impact on the first three quartiles, ranging from approximately 1–5% increase in registration probability. Conversely, the kiwi and dog attack messages had a negative impact on the highest quartile, at roughly 7–9% decrease. Areas with high levels of existing registration might be at their “saturation” point, where most people who could register do—and hence the messages would be expected to have minimal impact. This instead suggests that areas with high existing registration had a negative response to these two messages. This result is important for targeting but might also reflect other differences between the high registration areas and the other quartiles, which we explore further. It may also be the case that areas with higher registration rates have different sociodemographic or other characteristics. Although data is not available about owners, we further explore the available registration data in additional specifications below.

It is also worth noting that there are only minor differences between specifications (3) and (4), with the latter including post-code level fixed effects (the other regressions all cluster standard errors at the post-code level). The social nudge and loyalty messages did not yield significant effects.

**Fig. 9.**
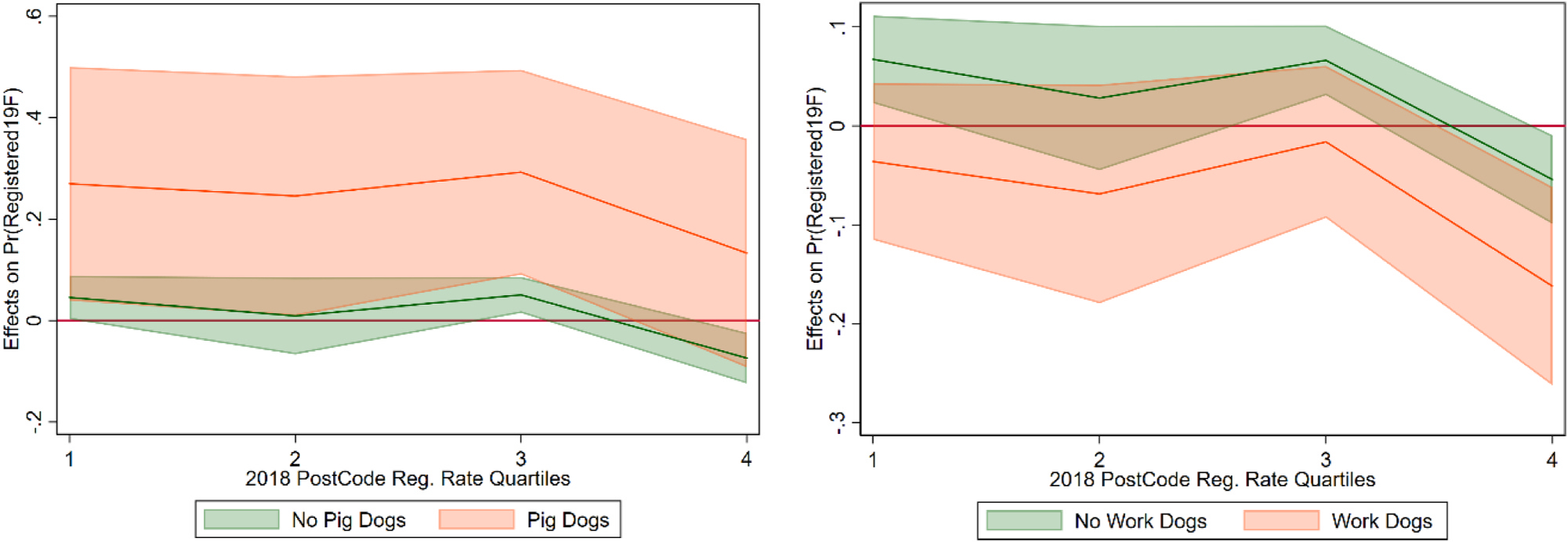
Marginal effect of kiwi message, with working dog and pig dog owners.

**Fig. 10.**
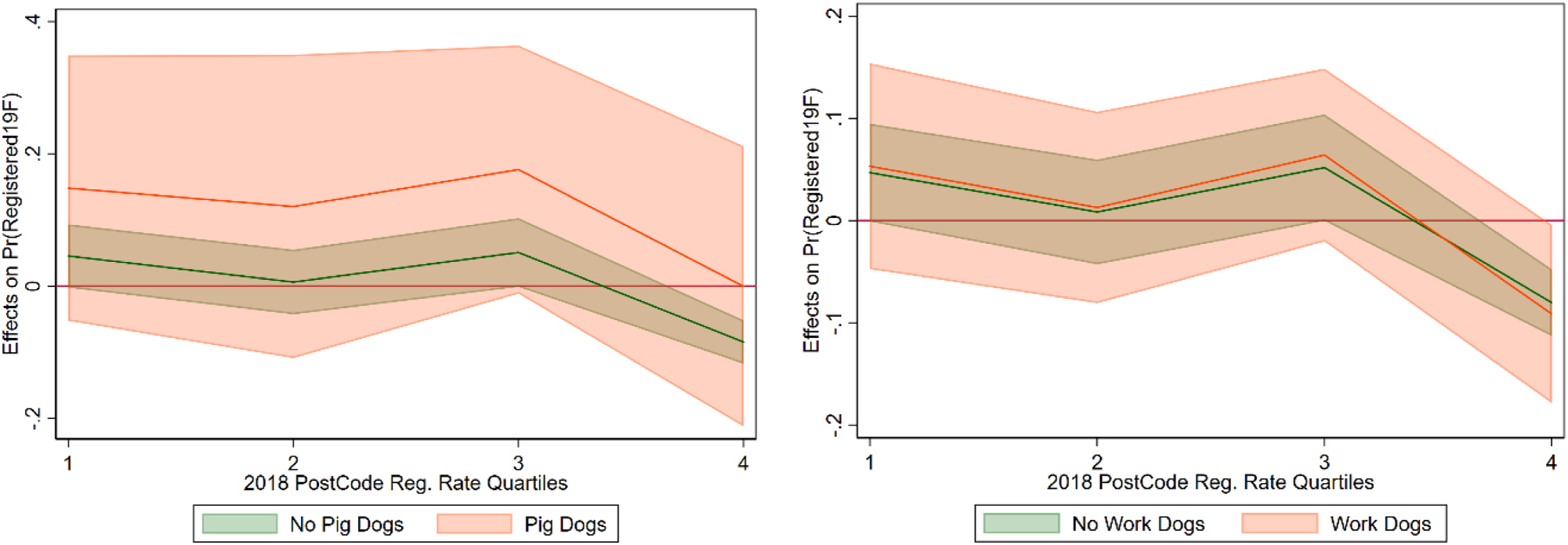
Marginal effect of dog attack message, with working dog and pig dog owners.

To analyze the impact of dog type on message effectiveness, we interact the working dog and pig dog variables with the message indicators. These models (Fig. 9 and Fig. 10) show that the kiwi message had a large and significant impact on pig dog owners in the first three quartiles, with an almost 30% increase in registration rates. There was not a significant difference for working dog owners, except for the highest registration quartile had a larger negative effect. The interactions between the dog attack message and dog types were insignificant.

**Fig. 11.**
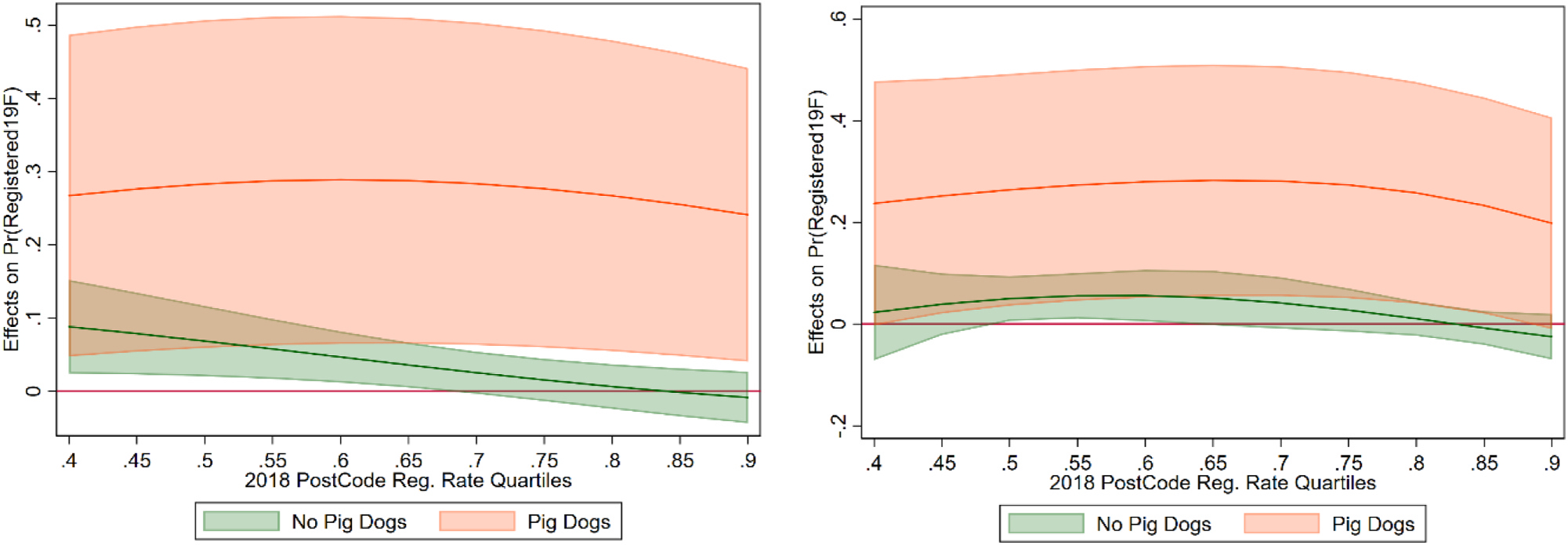
Marginal Effect of Kiwi Message and Pig Dogs, with Linear (left) and Quadratic (right) 2018 Post-Code Registration Rate Interactions.

Instead of using the quartiles, linear and quadratic interactions of the 2018 registration rate were also used (Fig. 11), with similar results. Those regressions also showed significant impacts of the kiwi message and dog attack messages, but not the other two. For dog types, the large effect on pig dog owners was consistent across specifications.

**Fig. 12.**
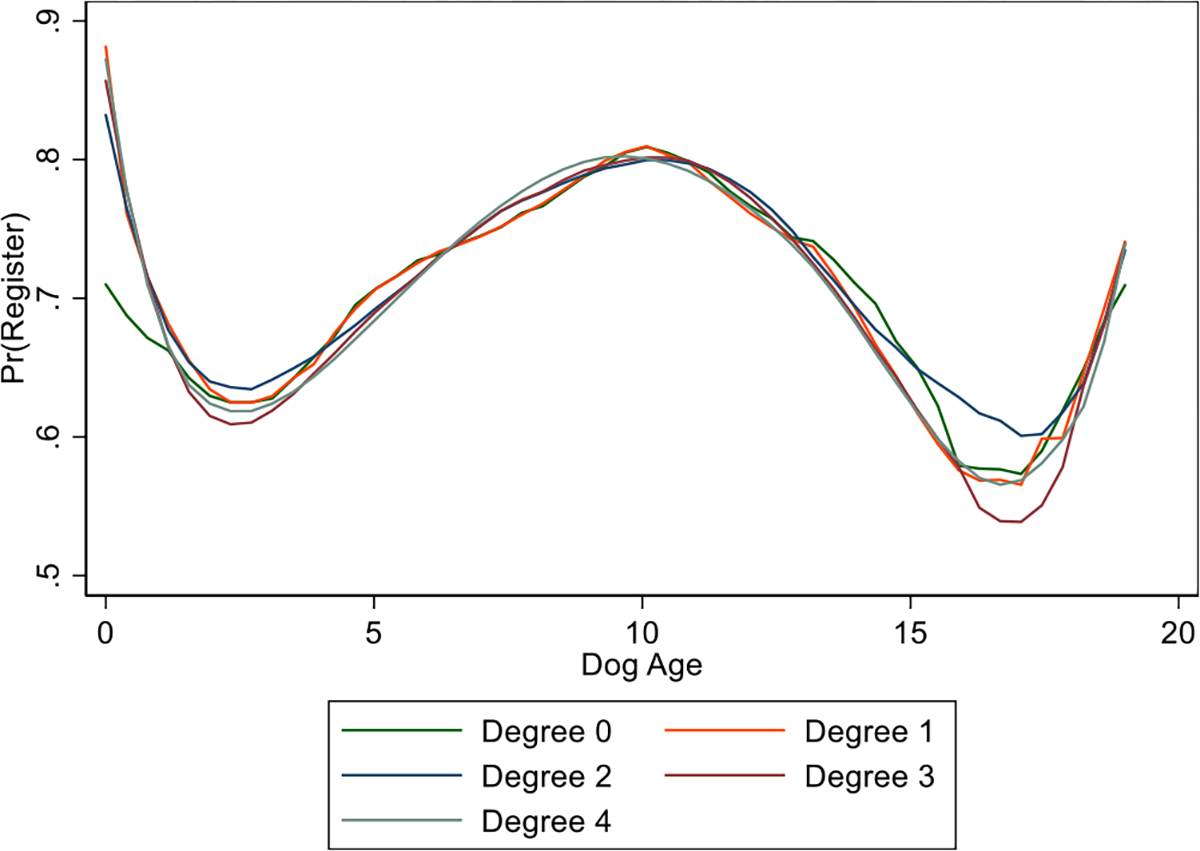
Local Polynomial Regressions, Dog Age. Notes: This figure shows the results of kernel-weighted local polynomial regressions of different degrees, using the Epchanikov kernel function.

It is also important to control for dog age for several reasons. One potential source of false negatives is dog deaths. Owners may be dropping out of the database due to dog deaths as opposed to non-compliance. One way to explore this effect is to see how registration rates vary with dog age, hypothesizing that older dogs would be less likely to be re-registered due to censoring. Since households can have multiple dogs, we use the maximum age among dogs in a household in our regressions.^19^ A first check of this hypothesis and any non-linear effects is carried out using local polynomial regression on registration and dog age. Fig. 12 shows the results of several different local polynomial regressions that vary by the degree of the polynomial. All of these regressions, which use the Epchanikov kernel function, produce a similar shape with three distinct break points. The figure shows decreasing registration from the first year to the third, then a rise to year 10 and a subsequent drop afterwards. The graph also shows a rise after approximately year 17. However, the data are quite thin at that age, with only 40 observations.

**Fig. 13.**
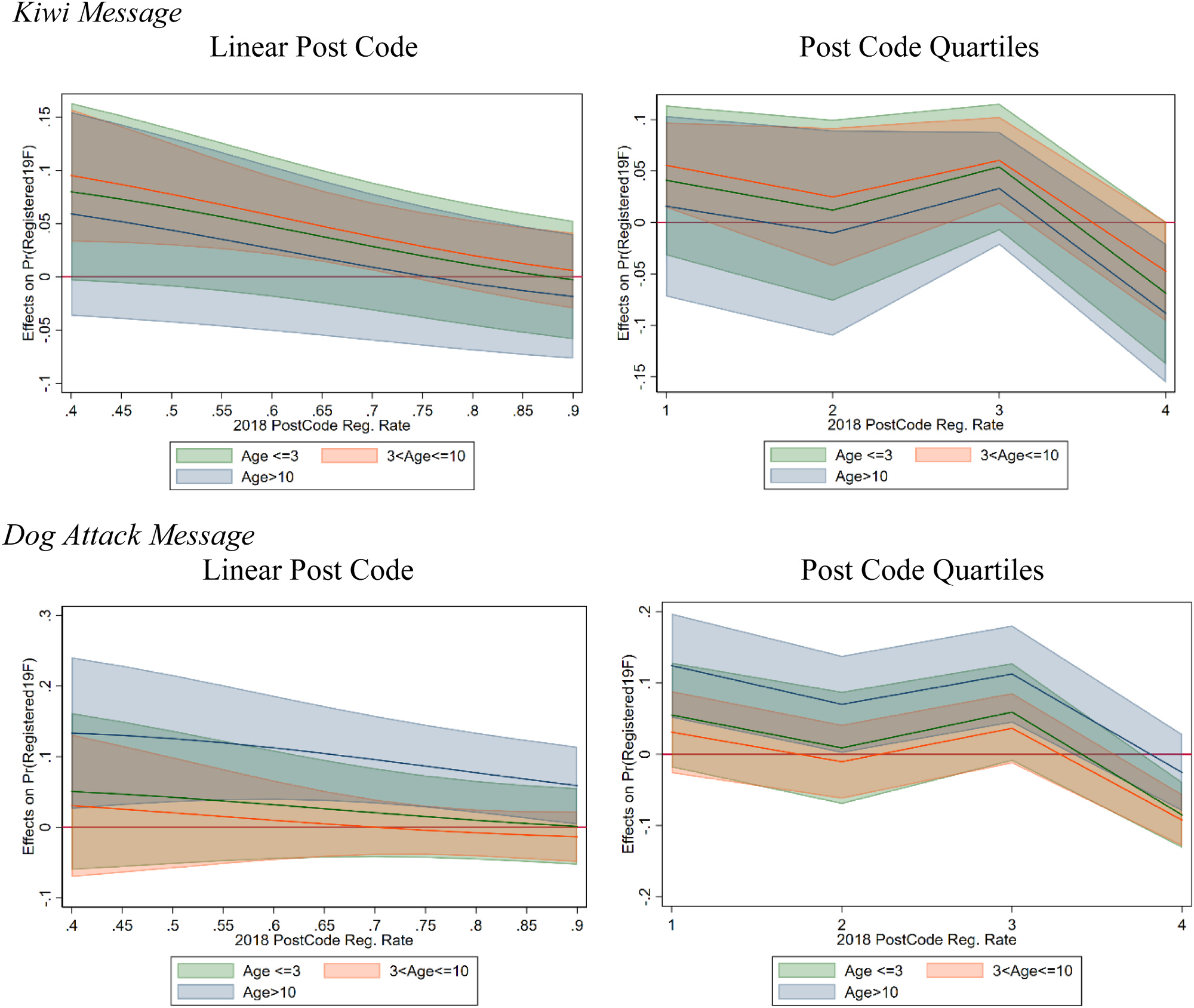
Marginal Effect of Kiwi and Dog Attack Messages, with Age Group and 2018 Post Code Registration Interactions.

Due to the consistent breakpoints shown by the local polynomial regressions, we create three indicators of dog age to be used in regressions: Dog Age <=3, 3 <Dog Age< =10, and Dog Age > 10. These variables are then used in regressions of the same format as above, but with interactions between the age dummies and the message types. The kiwi message (Fig. 13, first row) had a significant positive impact on dogs between 3 and 10 years of age, with positive but insignificant impacts on the other groups. The results are consistent with previous regressions across the 2018 post-code registration rates—the message impact declines in the higher 2018 registration areas. Conversely, the dog attack message is significant and positive in the group with dogs older than 10 (Fig. 13, second row). The loyalty and social comparison nudge did not have a significant impact on registration.

## 6. Discussion

Overall, the estimated regression results show significant heterogeneity in the impact of the messages across message types and households. There were only two messages (the dog attack and kiwi message) that had a significant impact, and only with certain groups. These two messages might therefore be deployed to promote more responsible behavior with dogs in areas with low registration rates, such as messages aimed at keeping dogs indoors or messages about local impacts on kiwi. Although a number of past papers have found that social-comparison based nudges motivated some behavioural change (Allcott and Rogers, 2014; Fanghella et al., 2019), there are still many areas where the utility of nudges has not been fully explored (John and Richardson, 2012). These results do not find a significant impact of the social comparison nudge.

This limited response to the social comparison message deserves further research, but reinforces some evidence that more action is needed in this area to motivate behaviour. In a summary of resistance to conservation movements, Holmes (2007) notes that acts of illegal hunting or farming can be implicit assertions of peoples’ longstanding right to do those activities. It is an easy form of protest to continue a banned activity, and interviews in New Zealand media about improper dog walking frequently encounter these kinds of opinions.^20^ The act of walking dogs off leash in a clearly signed park, or not registering a dog, is already going against social norms. Some people also might firmly believe that their dog would not kill a kiwi or other wildlife, so see the regulation as unjust (Kannemeyer, 2017; Kannemeyer et al., 2019). On the other hand, there may have been more effective ways to frame this particular message to better operationalize it. The message emphasized dogs registered, instead of owners themselves. The comparison group may not have been close enough to the recipients to elicit a full response (Bicchieri and Dimant, 2019). The messages were also difficult to personalize, without owner location or demographic information, which may have decreased effectiveness.

Our results suggest conservation messages should be targeted to maximize their impact. Pig dog owners, in particular, were found to be highly responsive to kiwi-based messages. This suggests that other kiwi-based engagement might also work with this group, such as kiwi aversion training for dogs.^21^ Respondents with dogs aged 3–10 years old were the most responsive to the kiwi message, whereas those with older dogs (>10 years) were most responsive to the dog attack message. Dog age might be used to target other conservation-related mailouts and future dog registration efforts. However, additional research is needed in this area. There was only limited demographic data available, and since dog age is correlated with other household characteristics, those other factors may be key drivers.

Conversely, areas that already had high registration rates were less responsive to the messages in this study. This may be because these areas are already near their maximum registration, given turnover. Conversely, there may be socioeconomic factors associated with these areas that are also correlated with the impact of messages, which should be explored in future research.

To demonstrate the impact of the messages on registration revenues, we predict the total revenue obtained with and without the messages using our econometric models and estimates of the registration cost for each household. These are therefore rough estimates of the potential gain in revenue for the council. Across the econometric specifications, the gain in revenue ranges from $1120 to $6450. The final model in Fig. 13, which accounts the widest set of variables, yields a gain in revenue of $6040. This small change of including printed messages into envelopes that were already going out therefore raised several thousand dollars for the council.

## 7. Conclusion

This research programme explores the impact of several different message types on dog owners in Northland. Since households with dogs can have a significant impact on local wildlife, it is important to identify which messages can be used to motivate behavior change. It is also generally important to improve dog registration rates for the council, as the registration information feeds into a number of planning and policy initiatives.

There is a lack of existing evidence-based research on conservation messaging (Kidd et al., 2019). Previous messaging research in other areas, like energy conservation (Allcott, 2011; Allcott and Rogers, 2014) and recreation (Allpress, 2019), has found that behavioural messaging techniques like nudging can improve environmental performance or behaviour. However, while environmentally sustainable behavior can both save people money and improve environmental performance, conservation-related actions typically cost the individual.

In our experiment on dog registration, four messages were developed with the local council and tested in a randomised control trial with several thousand participants. The messages were focussed on kiwi conservation, dog attacks, a social comparison nudge, and loyalty to your dog. Results found that only the kiwi message and dog attack message were effective at increasing registration rates, and only in areas with low registration rates the previous year.

Surprisingly, the social comparison /nudge message was not found to be effective in this experiment. Social pressure may not be effective with some activities that cause mental or tangible costs on people, especially if action is intertwined with a other cultural and historical factors (Kannemeyer et al., 2019). The loyalty message did not induce additional registrations either. Similar messaging was used as part of wider campaign that successfully increased dog neutering/spaying. However, that campaign included a number of other outreach activities, free vet services, and hip hop-based theming. The loyalty message alone may therefore not be sufficient to change behaviour.

The results from this study also illustrate the importance of targeting in message design. Several identifiable characteristics can be used to better target respondents, including dog age, post code registration rate, and dog types. For example, pig dog owners were found to have a 90% increase in registration rates after receiving the kiwi-focussed message. Those households may therefore be more open to other kiwi-related outreach like kiwi aversion training. Since pig dog hunting involves loose dogs in areas with kiwi, additional engagement with these owners represents an important step forward. Overall, results suggest that targeting areas with low registration rates should be beneficial. This also highlights the need to examine behavior in those areas for future conservation work.

Although tested in Northern New Zealand, the characteristics of this conservation problem should make the results relevant for other areas. Conservation messaging is widely used with the intent to motivate action, so it is important to empirically test effectiveness. While the present study uses contemporary techniques in a large RCT, additional research is needed in this area. Other studies have found that nudge effectiveness can depend on other factors such as political ideology (Costa and Kahn, 2013) and other factors (Kuehnhanss, 2019), which should be a topic of future research.

## Figures and Tables

**Table 1:** Messaging summary statistics.

Messages	N	% Register

Control (No Message)	1887	71.7
Treatment (Any Message)	4234	72.1
Treatment, by Message		
Dog Attacks	1063	72.4
Kiwi	1049	73.4
Social Comparison/Nudge	1051	72.1
Loyalty	1071	70.5

**Table 2:** Owner/dog summary statistics.

Variable	Mean	Std. Dev.	Min	Max

Any Work Dogs	0.199	0.399	0	1
Any Pig Dogs	0.030	0.170	0	1
Any Pet Dogs	0.792	0.406	0	1
Total Reg. Cost	76.029	47.050	0	510
Total Dogs	1.471	0.924	1	9
Postal Reg. Rate 2018	0.779	0.105	0.439	0.904
Age Primary Dog	6.968	4.054	0	19

**Table 3:** Summary statistics (mean and standard deviation) across treatments.

Variable	Control	Dog Attack	Kiwi	Social	Loyalty

Any Work Dogs	0.184 (0.388)	0.182 (0.386)	0.211 (0.408)	0.200 (0.400)	0.200 (0.403)
Any Pig Dogs	0.028 (0.164)	0.025 (0.157)	0.026 (0.158)	0.028 (0.164)	0.028 (0.180)
Any Pet Dogs	0.807 (0.395)	0.809 (0.393)	0.780 (0.415)	0.792 (0.406)	0.792 (0.414)
Total Reg. Cost	72.194 (40.185)	74.685 (44.496)	75.072 (42.792)	75.233 (45.440)	75.233 (43.246)
Total Dogs	1.395 (0.791)	1.437 (0.847)	1.452 (0.840)	1.457 (0.896)	1.457 (0.860)
Postal Reg. Rate 2018	0.780 (0.103)	0.779 (0.105)	0.779 (0.104)	0.776 (0.108)	0.776 (0.107)
Age Primary Dog	6.850 (3.939	6.731 (3.893)	6.940 (4.158)	7.194 (4.294)	7.194 (4.008)

Note: Standard Deviations appear in parentheses

**Table 4:** Messaging summary statistics for pig dog and working dog owners.

	Working Dog Owners		Pig Dog Owners	
Message	% Register	N	% Register	N

Control (No Message)	81.3	348	48.1	52
Dog Attack	85.0	193	55.6	27
Kiwi	79.6	221	77.8	27
Social Comparison/Nudge	81.4	210	51.7	29
Loyalty	80.7	218	55.6	36

**Table 5:** Econometric results with post code quartile interactions.

	(1)	(2)	(3)	(4)

Quartile 1#DogAttackMessage	0.4097 *** (0.09669)	0.4317 *** (0.09515)	0.4585 *** (0.1114)	0.4369 *** (0.1096)
Quartile 2#DogAttackMessage	0.3703 *** (0.1135)	0.3439 *** (0.1138)	0.3537 *** (0.1241)	0.3608 *** (0.1237)
Quartile 3#DogAttackMessage	0.4813 *** (0.1301)	0.5077 *** (0.1291)	0.5094 *** (0.1345)	0.5102 *** (0.1391)
Quartile 1#KiwiMessage	0.4177 *** (0.1186)	0.4330 *** (0.1195)	0.4116 *** (0.1336)	0.4024 *** (0.1338)
Quartile 2#KiwiMessage	0.2968 * (0.1654)	0.2832 (0.1744)	0.2813 (0.1932)	0.2818 (0.1903)
Quartile 3#KiwiMessage	0.3613 *** (0.1371)	0.3814 *** (0.1272)	0.4390 *** (0.1334)	0.4234 *** (0.1333)
Quartile 1#SocialMessage	0.2332 (0.1702)	0.2508 (0.1649)	0.1813 (0.1699)	0.1892 (0.1849)
Quartile 2#SocialMessage	0.1974 (0.1405)	0.1921 (0.1389)	0.1411 (0.1447)	0.1559 (0.1513)
Quartile 3#SocialMessage	0.3372 ** (0.1620)	0.3442 ** (0.1635)	0.2674 (0.1687)	0.2623 (0.1758)
Quartile 1#LoyaltyMessage	0.2132 (0.1686)	0.2303 (0.1612)	0.1691 (0.1514)	0.1545 (0.1485)
Quartile 2#LoyaltyMessage	0.01080 (0.1628)	−0.01153 (0.1664)	−0.01344 (0.1535)	−0.009162 (0.1486)
Quartile 3#LoyaltyMessage	0.1643 (0.1860)	0.1478 (0.1743)	0.09577 (0.1811)	0.1014 (0.1764)
Dog Attack Message	−0.3155 *** (0.06755)	−0.3197 *** (0.06389)	−0.3181 *** (0.06734)	−0.3153 *** (0.06655)
Kiwi Message	−0.2402 ** (0.09489)	−0.2563 *** (0.09442)	−0.2543 ** (0.1100)	−0.2638 ** (0.1076)
Social Message	−0.1834 * (0.1079)	−0.1941 * (0.1047)	−0.1527 (0.1032)	−0.1582 (0.1089)
Loyalty Message	−0.1501 (0.1231)	−0.1513 (0.1137)	−0.1323 (0.1091)	−0.1412 (0.1032)
Quartile 1: 2018 Post Code Rate	−0.9881 *** (0.09927)	−1.0071 *** (0.08717)	−0.8684 *** (0.08470)	0.02349 (0.07407)
Quartile 2: 2018 Post Code Rate	−0.4373 *** (0.09600)	−0.4680 *** (0.09411)	−0.4195 *** (0.09799)	−0.2534 *** (0.07944)
Quartile 3: 2018 Post Code Rate	−0.4542 *** (0.09337)	−0.5085 *** (0.09170)	−0.4398 *** (0.09581)	−0.3922 *** (0.07699)
Any Pet Dogs		0.2601 ** (0.1047)	0.001065 (0.1003)	−0.02969 (0.09633)
Any Work Dogs		0.6014 *** (0.1187)	−0.2741 ** (0.1071)	−0.3236 *** (0.1093)
Any Pig Dogs		−0.1685 (0.1229)	−0.8620 *** (0.1290)	−0.8919 *** (0.1269)
Total Cost			−0.05712 *** (0.003870)	−0.05676 *** (0.003942)
Total Dogs			3.1715 *** (0.2197)	3.1508 *** (0.2213)
Constant	1.0978 *** (0.06374)	0.8163 *** (0.1238)	0.8806 *** (0.1182)	0.7110 *** (0.09428)
Observations	6121	6121	6121	6121
Fixed Effects				Yes

*** p < 0.01

** p < 0.05

* p < 0.1

Robust standard errors in parentheses

**Fig. A1.**
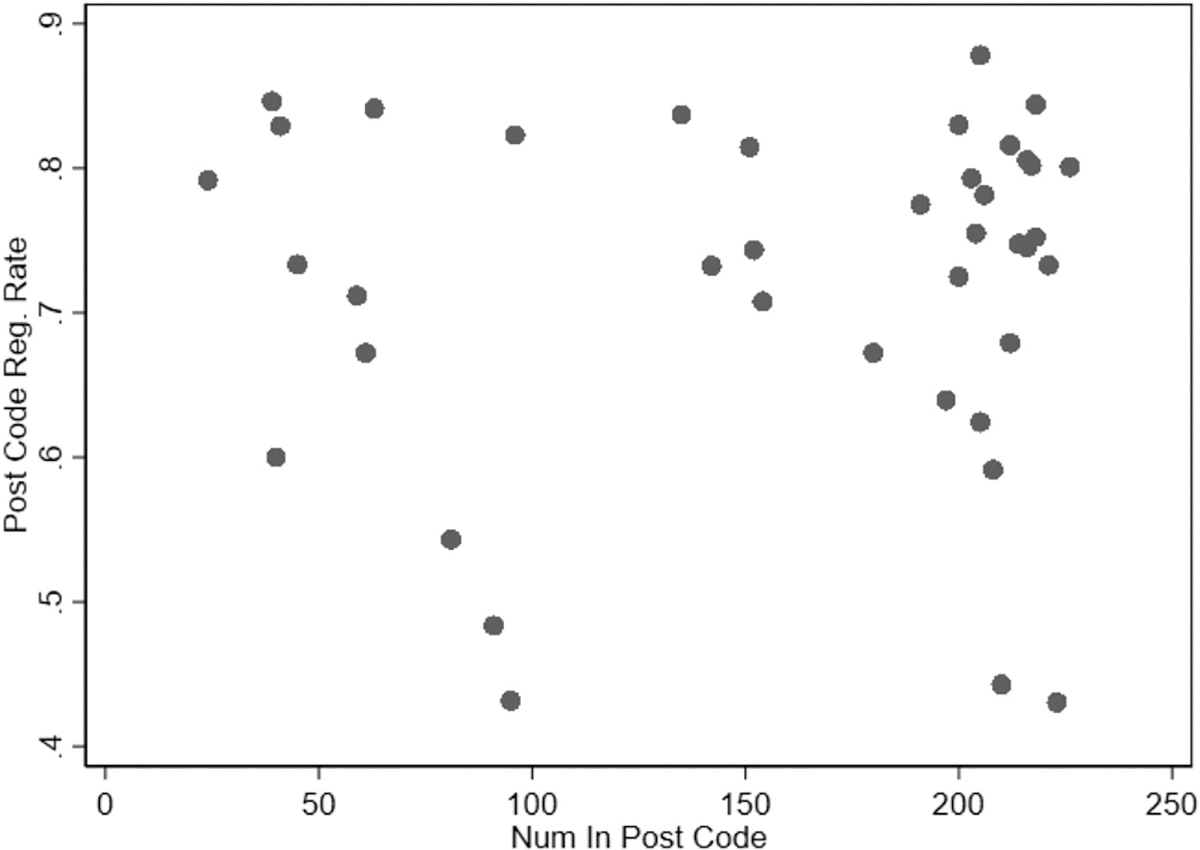
Number in Post Code and Registration Rate.

**Fig. A2.**
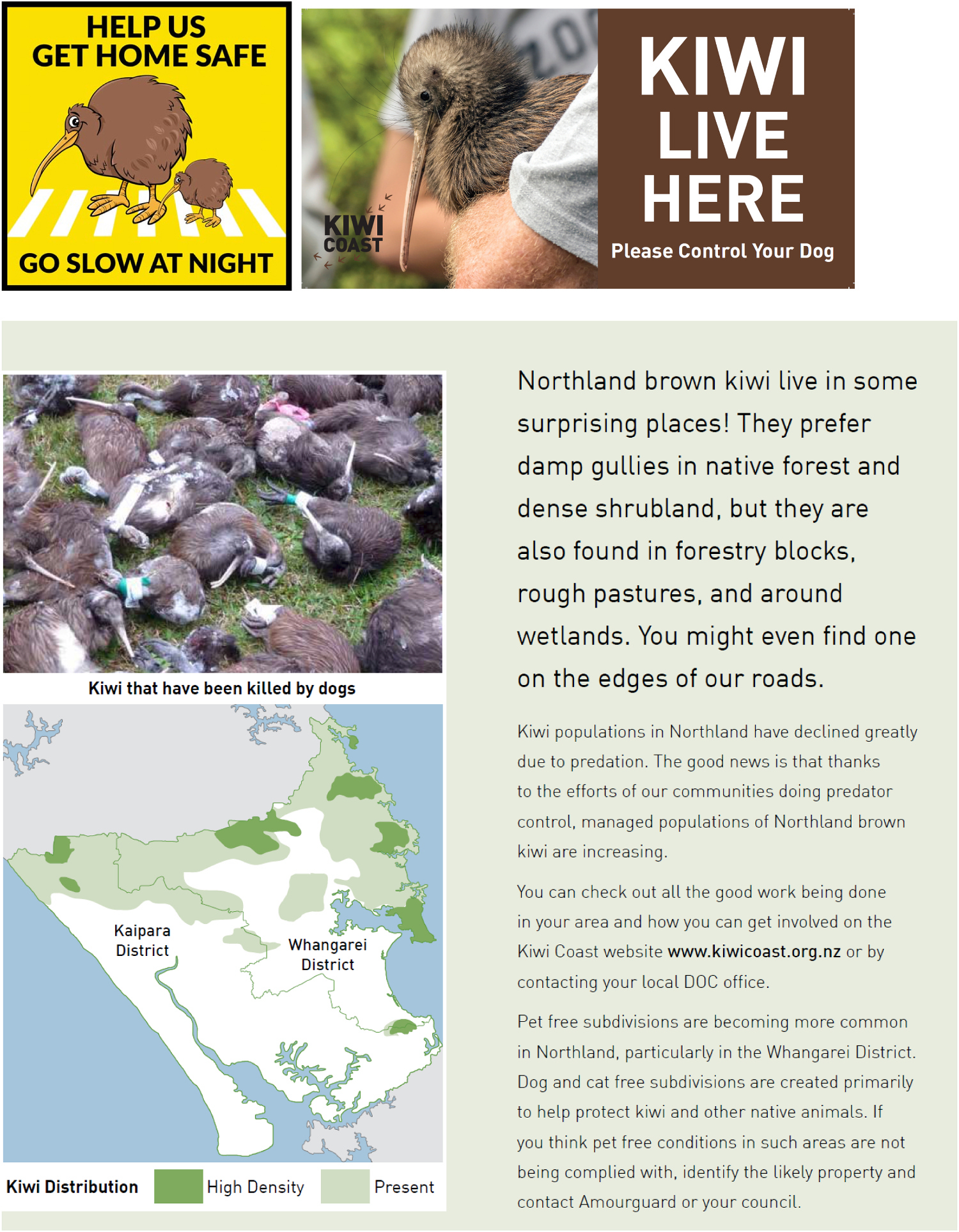
Additional Examples of Existing Messaging and Pamphlets.

## Footnotes

1 Additional information can be found at https://www.doc.govt.nz/nature/native-animals/birds/birds-a-z/kiwi/facts/, and https://www.stuff.co.nz/auckland/local-news/northland/105195568/kiwi-killed-by-dog-in-bay-of-islands, accessed May, 2020.

2 The problem of dogs off leash regularly appears in local media: https://www.nzherald.co.nz/animal-welfare/news/article.cfm?c_id=119&objectid=12273760, https://www.nzherald.co.nz/nz/news/article.cfm?c_id=1&objectid=12265870.

3 Personal communication with Kiwis for kiwi, regarding fundraising efforts, May 2017.

4 For some recent examples, see (New Zealand) https://dpmc.govt.nz/our-programmes/policy-project/policy-methods-toolbox/behavioural-insights, and (UK) https://www.bi.team/publications/behavioural-government/

5 Summary Census figures can be found at: https://www.stats.govt.nz/tools/2018-census-place-summaries/northland-region

6 Examples include https://www.nzherald.co.nz/nz/news/article.cfm?c_id=1&objectid=12180950, and https://www.rnz.co.nz/news/national/409707/dog-owner-charged-over-kiwi-death.

7 See, for instance, this news article on kiwi kills: https://www.stuff.co.nz/auckland/local-news/northland/105195568/kiwi-killed-by-dog-in-bay-of-islands

8 Obtained from a 2020 public records request to https://www.acc.co.nz/ and the Department of Internal Affairs’s Dog Registration Database.

9 An example of a local government pamphlet distributed at tourist areas and libraries can be found here: https://www.kiwicoast.org.nz/wp-content/uploads/2017/01/Kiwi-DogOwners-Brochure.pdf

10 For example, the 2018 update to the Dog Control Bylaw took several years and multiple rounds of negotiations https://www.nzherald.co.nz/northern-advocate/news/far-north-district-council-receives-record-submissions-on-dog-control-bylaw/DMXHU6UKOO5ADDFA36IT3P2GRU/.

11 A good resource for applying and designing behavioural insights is through BIT’s EAST approach (easy, attractive, social, and timely). For additional information, see https://www.behaviouralinsights.co.uk/wp-content/uploads/2015/07/BIT-Publication-EAST_FA_WEB.pdf.

12 For example, https://www.influenceatwork.com/principles-of-persuasion/#reciprocity.

13 Due to some data entry, coding, and related errors between the dog database and owner records, some observations were discarded.

14 We also do not know the postal codes for previous years. Appendix Fig. 1 contains a graph of the number of dog owners in each post code plotted with the post-code registration rate.

15 There are several potential reasons for the decrease. First, 2018 was a higher registration year than usual, with 2017 registration rates more similar to 2019. Additionally, there was a contentious public debate about a new dog control bylaw in the area, characterised by protests and the spread of misinformation about the local government. A summary can be found at https://www.nzherald.co.nz/northern-advocate/news/article.cfm?c_id=1503450&objectid=12136773.

16 The FNDC 2018 Dog Management Policy contains more information on these definitions and how they are regulated.

17 For more information on pig dog hunting, see https://teara.govt.nz/en/hunting/page-5

18 The impact of messages on registration was theorized to vary over previous year registration rates. The quadratic specification is used to test a non-linear impact of post-code registration. Additional polynomials were also explored. While they produced similar marginal effects, the quadratic had the best fit, in terms of pseudo R^2^.

19 Age of the primary dog was also used, with similar results.

20 For example, “It’s a good call to try to save the birds and everything but as far as me walking my dog in the morning, yeh I’m not interested in anybody telling me I can’t.” https://www.rnz.co.nz/news/national/378218/call-for-ban-on-dog-walkers-along-coastlines-with-little-penguins, also see https://www.rnz.co.nz/news/national/383715/dog-owners-urged-to-control-pets-after-native-animals-killed.

21
https://www.kiwisforkiwi.org/what-we-do/how-were-saving-kiwi/avoidance-training-for-dogs/

## Appendix

see Fig. A1 and Fig. A2.

**Table A1:** Probit results – full population.

Variable	(1)	(2)	(3)

AnyMessage	0.01110 (0.03827)	0.01404 (0.04651)	0.2490 (0.1698)
Any Pet Dogs	0.1256 (0.08428)	0.05593 (0.1000)	0.2004 (0.1845)
Any Work Dogs	0.6902 *** (0.08411)	0.5776 *** (0.1137)	0.7375 *** (0.1605)
Any Pig Dogs	−0.06364 (0.1164)	−0.1103 (0.1277)	−0.2090 (0.2427)
Any Neuter Dogs	0.8179 *** (0.03734)	0.7608 *** (0.04604)	0.7563 *** (0.07791)
Any Message*Any Pet Dogs			−0.2452 (0.1784)
Any Message*Any Work Dogs			−0.2680 * (0.1505)
Any Message*Any Pig Dogs			0.1330 (0.2743)
Any Message*Any Neuter Dogs			−0.004659 (0.08451)
Zip Reg. Rate 2018		2.3329 *** (0.2672)	−0.9011 *** (0.1098)
Constant	−0.1425 (0.08906)	−1.8345 *** (0.2200)	0.6160 *** (0.2047)
Zip Code FE	No	No	Yes
Observations	6121	6121	6121

*** p < 0.01

** p < 0.05

* p < 0.1.

## References

[R1] Allcott H, 2011. Social norms and energy conservation. J. Public Econ. 95 (9), 1082–1095.

[R2] Allcott H, Rogers T, 2014. The short-run and long-run effects of behavioral interventions: experimental evidence from energy conservation. Am. Econ. Rev. 104 (10), 3003–3037.

[R3] Allpress J, 2019. Nudging’ Visitors to Notice Safeswim Signs. Auckland Council. Research and Evaluation Unit, Auckland New Zealand.

[R4] Banatoski I, Dellaripa B, Hires S, Naidoo L, Rooney E, 2017. The Role of Public Perceptions in Reducing Risks to Coastal Wildlife from Interactions with Dogs. Department of Conservation and Worcester Polytechnic Institute. Marine Species and Threats Team.

[R5] Barr S, Shaw G, Coles T, 2011. Times for (Un)sustainability? Challenges and opportunities for developing behaviour change policy. A case-study of consumers at home and away. Glob. Environ. Change 21 (4), 1234–1244.

[R6] Bennett NJ, Roth R, Klain SC, Chan K, Christie P, Clark DA, Cullman G, Curran D, Durbin TJ, Epstein G, Greenberg A, Nelson MP, Sandlos J, Stedman R, Teel TL, Thomas R, Veríssimo D, Wyborn C, 2017. Conservation social science: Understanding and integrating human dimensions to improve conservation. Biol. Conserv. 205, 93–108.

[R7] Bernedo M, Ferraro PJ, Price M, 2014. The persistent impacts of norm-based messaging and their implications for water conservation. J. Consum. Policy 37 (3), 437–452.

[R8] Bicchieri C, Dimant E, 2019. Nudging with care: the risks and benefits of social information. Public Choice.

[R9] Byerly H, Balmford A, Ferraro PJ, Hammond Wagner C, Palchak E, Polasky S, Ricketts TH, Schwartz AJ, Fisher B, 2018. Nudging pro-environmental behavior: evidence and opportunities. Front. Ecol. Environ. 16 (3), 159–168.

[R10] Christakis NA, Fowler JH, 2008. The collective dynamics of smoking in a large social network. N. Engl. J. Med. 358 (21), 2249–2258. 18499567 10.1056/NEJMsa0706154 PMC2822344

[R11] Congiu L, Moscati I, 2020. Message and environment: a framework for nudges and choice architecture. Behav. Public Policy 4 (1), 71–87.

[R12] Costa DL, Kahn ME, 2013. Energy conservation “nudges” and environmentalist ideology: evidence from a randomized residential electricity field experiment. J. Eur. Econ. Assoc. 11 (3), 680–702.

[R13] Dale AR, Statham S, Podlesnik CA, Elliffe D, 2013. The acquisition and maintenance of dogs’ aversion responses to kiwi (Apteryx spp.) training stimuli across time and locations. Appl. Anim. Behav. Sci. 146 (1), 107–111.

[R14] Ebeling F, Lotz S, 2015. Domestic uptake of green energy promoted by opt-out tariffs. Nat. Clim. Change 5 (9), 868–871.

[R15] Eppink F, Walsh PJ, MacDonald E, 2021. Demographic and psychographic drivers of public acceptance of novel invasive pest control technologies. Ecol. Soc. 26 (1), art31.

[R16] Fanghella V, d’Adda G, Tavoni M, 2019. On the use of nudges to affect spillovers in environmental behaviors. Front. Psychol 10 (61). 10.3389/fpsyg.2019.00061 PMC6362870 30761038

[R17] Goldstein NJ, Cialdini RB, Griskevicius V, 2008. A room with a viewpoint: using social norms to motivate environmental conservation in hotels. J. Consum. Res. 35 (3), 472–482.

[R18] Hallsworth M, Snijders V, Burd H, Prestt J, Judah G, Huf S, Halpern D, 2016. Applying Behavioural Insights: Simple Ways to Improve Health Outcomes. Report of the WISH Behavioral Insights Forum 2016. World Innovation Summit for Health.

[R19] Haynes L, Service O, Goldacre B, Torgerson D, 2012. Test, Learn, Adapt: Developing Public Policy with Rnadomised Control Trials. UK Cabinet Office Behavioural Insignts Team.

[R20] Holmes G, 2007. Protection, politics and protest: understanding resistance to conservation. Conserv. Soc. 5 (2), 184–201.

[R21] Jachimowicz JM, Hauser OP, O’Brien JD, Sherman E, Galinsky AD, 2018. The critical role of second-order normative beliefs in predicting energy conservation. Nat. Human Behav. 2 (10), 757–764. 31406290 10.1038/s41562-018-0434-0

[R22] James B, 2001. Evaluation of kiwi advocacy programmes in northland and coromandel. Department of conservation Te papa atawhai. Wellingt. Sci. Conserv. 161.

[R23] Janz NK, Becker MH, 1984. The health belief model: a decade later. Health Educ. Q. 11 (1), 1–47. 6392204 10.1177/109019818401100101

[R24] John P, Richardson L, 2012. Nudging Citizens Towards Localism? The British Academy. London, Policy Centre.

[R25] Kannemeyer R, 2017. A systematic literature review of attitudes to pest control methods in New Zealand. New Zealand’s Biological Heritage National Science Challenge. Reducing risks and threats programme, Manaaki Whenua.

[R26] Kannemeyer R, Walsh P, Greenaway A, 2019. Enhancing control of dogs preying on kiwi: using Q methodology to understand viewpoints in Northland Manaaki Whenua-Landcare Research. Auckland. Contract Report LCR 3482.

[R27] Kidd LR, Garrard GE, Bekessy SA, Mills M, Camilleri AR, Fidler F, Fielding KS, Gordon A, Gregg EA, Kusmanoff AM, Louis W, Moon K, Robinson JA, Selinske MJ, Shanahan D, Adams VM, 2019. Messaging matters: a systematic review of the conservation messaging literature. Biol. Conserv. 236, 92–99.

[R28] Kondylis F, Mueller V, Sheriff G, Zhu S, 2016. Do female instructors reduce gender bias in diffusion of sustainable land management techniques? Experimental evidence from mozambique. World Dev. 78, 436–449.

[R29] Kormos C, Gifford R, Brown E, 2015. The influence of descriptive social norm information on sustainable transportation behavior:a field experiment. Environ. Behav. 47 (5), 479–501.

[R30] Kubo T, Shoji Y, Tsuge T, Kuriyama K, 2018. Voluntary contributions to hiking trail maintenance: evidence from a field experiment in a national park, Japan. Ecol. Econ. 144, 124–128.

[R31] Kuehnhanss CR, 2019. The challenges of behavioural insights for effective policy design. Policy Soc. 38 (1), 14–40.

[R32] Kusmanoff AM, Fidler F, Gordon A, Garrard GE, Bekessy SA, 2020. Five lessons to guide more effective biodiversity conservation message framing. Conserv. Biol. 34, 1131–1141. 32043648 10.1111/cobi.13482

[R33] MacDonald E, Milfont T, Gavin M, 2015. What drives cat-owner behaviour? First steps towards limiting domestic-cat impacts on native wildlife. Wildl. Res 42 (3), 257–265.

[R34] McLeod LJ, Hine DW, Please PM, Driver AB, 2015. Applying behavioral theories to invasive animal management: towards an integrated framework. J. Environ. Manag. 161, 63–71. 10.1016/j.jenvman.2015.06.048 26151198

[R35] Momsen K, Stoerk T, 2014. From intention to action: can nudges help consumers to choose renewable energy? Energy Policy 74, 376–382.

[R36] Moussaoui LS, Desrichard O, Milfont TL, 2019. Do environmental prompts work the same for everyone? A test of environmental attitudes as a moderator. Front. Psychol. 10, 3057, 3057–3057. 32116871 10.3389/fpsyg.2019.03057 PMC7015073

[R37] Rogers RW, 1985. Attitude change and information integration in fear appeals. Psychol. Rep. 56 (1), 179–182.

[R38] St John FAV, Edwards-Jones G, Jones JPG, 2010. Conservation and human behaviour: lessons from social psychology. Wildl. Res. 37 (8), 658–667.

[R39] Thaler RH, Sunstein CR, 2008. Nudge: Improving Decisions About Health, Wealth, and Happiness. Yale University Press, New Haven, CT, US.

[R40] Villatoro FJ, Naughton-Treves L, Sepúlveda MA, Stowhas P, Mardones FO, Silva-Rodríguez EA, 2019. When free-ranging dogs threaten wildlife: public attitudes toward management strategies in southern Chile. J. Environ. Manag. 229, 67–75. 10.1016/j.jenvman.2018.06.035 30143314

